# Large eQTL meta-analysis reveals differing patterns between cerebral cortical and cerebellar brain regions

**DOI:** 10.1038/s41597-020-00642-8

**Published:** 2020-10-12

**Authors:** Solveig K. Sieberts, Thanneer M. Perumal, Minerva M. Carrasquillo, Mariet Allen, Joseph S. Reddy, Gabriel E. Hoffman, Kristen K. Dang, John Calley, Philip J. Ebert, James Eddy, Xue Wang, Anna K. Greenwood, Sara Mostafavi, The CommonMind Consortium (CMC), Schahram Akbarian, Jaroslav Bendl, Michael S. Breen, Kristen Brennand, Leanne Brown, Andrew Browne, Joseph D. Buxbaum, Alexander Charney, Andrew Chess, Lizette Couto, Greg Crawford, Olivia Devillers, Bernie Devlin, Amanda Dobbyn, Enrico Domenici, Michele Filosi, Elie Flatow, Nancy Francoeur, John Fullard, Sergio Espeso Gil, Kiran Girdhar, Attila Gulyás-Kovács, Raquel Gur, Chang-Gyu Hahn, Vahram Haroutunian, Mads Engel Hauberg, Laura Huckins, Rivky Jacobov, Yan Jiang, Jessica S. Johnson, Bibi Kassim, Yungil Kim, Lambertus Klei, Robin Kramer, Mario Lauria, Thomas Lehner, David A. Lewis, Barbara K. Lipska, Kelsey Montgomery, Royce Park, Chaggai Rosenbluh, Panagiotis Roussos, Douglas M. Ruderfer, Geetha Senthil, Hardik R. Shah, Laura Sloofman, Lingyun Song, Eli Stahl, Patrick Sullivan, Roberto Visintainer, Jiebiao Wang, Ying-Chih Wang, Jennifer Wiseman, Eva Xia, Wen Zhang, Elizabeth Zharovsky, The AMP-AD Consortium, Laura Addis, Sadiya N. Addo, David Charles Airey, Matthias Arnold, David A. Bennett, Yingtao Bi, Knut Biber, Colette Blach, Elizabeth Bradhsaw, Paul Brennan, Rosa Canet-Aviles, Sherry Cao, Anna Cavalla, Yooree Chae, William W. Chen, Jie Cheng, David Andrew Collier, Jeffrey L. Dage, Eric B. Dammer, Justin Wade Davis, John Davis, Derek Drake, Duc Duong, Brian J. Eastwood, Michelle Ehrlich, Benjamin Ellingson, Brett W. Engelmann, Sahar Esmaeelinieh, Daniel Felsky, Cory Funk, Chris Gaiteri, Samuel Gandy, Fan Gao, Opher Gileadi, Todd Golde, Shaun E. Grosskurth, Rishi R. Gupta, Alex X. Gutteridge, Vahram Haroutunian, Basavaraj Hooli, Neil Humphryes-Kirilov, Koichi Iijima, Corey James, Paul M. Jung, Rima Kaddurah-Daouk, Gabi Kastenmuller, Hans-Ulrich Klein, Markus Kummer, Pascale N. Lacor, James Lah, Emma Laing, Allan Levey, Yupeng Li, Samantha Lipsky, Yushi Liu, Jimmy Liu, Zhandong Liu, Gregory Louie, Tao Lu, Yiyi Ma, Yasuji Y. Matsuoka, Vilas Menon, Bradley Miller, Thomas P. Misko, Jennifer E. Mollon, Kelsey Montgomery, Sumit Mukherjee, Scott Noggle, Ping-Chieh Pao, Tracy Young Pearce, Neil Pearson, Michelle Penny, Vladislav A. Petyuk, Nathan Price, Danjuma X. Quarless, Brinda Ravikumar, Janina S. Ried, Cara Lee Ann Ruble, Heiko Runz, Andrew J. Saykin, Eric Schadt, James E. Scherschel, Nicholas Seyfried, Joshua M. Shulman, Phil Snyder, Holly Soares, Gyan P. Srivastava, Henning Stockmann, Mariko Taga, Shinya Tasaki, Jessie Tenenbaum, Li-Huei Tsai, Aparna Vasanthakumar, Astrid Wachter, Yaming Wang, Hong Wang, Minghui Wang, Christopher D. Whelan, Charles White, Kara H. Woo, Paul Wren, Jessica W. Wu, Hualin S. Xi, Bruce A. Yankner, Steven G. Younkin, Lei Yu, Maria Zavodszky, Wenling Zhang, Guoqiang Zhang, Bin Zhang, Jun Zhu, Larsson Omberg, Mette A. Peters, Benjamin A. Logsdon, Philip L. De Jager, Nilüfer Ertekin-Taner, Lara M. Mangravite

**Affiliations:** 1 Sage Bionetworks, Seattle, WA 98121 USA; 2 Department of Neuroscience, Mayo Clinic Florida, Jacksonville, FL 32224 USA; 3 Pamela Sklar Division of Psychiatric Genomics, Department of Genetics and Genomic Sciences, Icahn School of Medicine at Mount Sinai, New York, NY 10029 USA; 4 Icahn Institute for Data Science and Genomic Technology, Department of Genetics and Genomic Sciences, Icahn School of Medicine at Mount Sinai, New York, NY 10029 USA; 5 Lilly Research Labs, Eli Lilly and Company, Indianapolis, IN 46225 USA; 6 Departments of Statistics and Medical Genetics, University of British Columbia, Vancouver, British Columbia Canada; 7 Centre for Molecular Medicine and Therapeutics, Vancouver, British Columbia Canada; 8 Canadian Institute for Advanced Research, CIFAR Program in Child and Brain Development, Toronto, Ontario Canada; 9 Center for Translational & Computational Neuroimmunology, Department of Neurology, Columbia University Medical Center, New York, NY 10032 USA; 10 Department of Neurology, Mayo Clinic Florida, Jacksonville, FL 32224 USA; 11 Division of Psychiatric Epigenomics, Department of Psychiatry, Icahn School of Medicine at Mount Sinai, New York, New York USA; 12 Division of Psychiatric Genomics, Department of Psychiatry, Icahn School of Medicine at Mount Sinai, New York, New York USA; 13 Seaver Autism for Research and Treatment, Department of Psychiatry, Icahn School of Medicine at Mount Sinai, New York, New York USA; 14 Friedman Brain Institute, Icahn School of Medicine at Mount Sinai, New York, New York USA; 15 Institute for Genomics and Multiscale Biology, Department of Genetics and Genomic Sciences, Icahn School of Medicine at Mount Sinai, New York, New York USA; 16 Department of Cell, Developmental and Regenerative Biology, Icahn School of Medicine at Mount Sinai, New York, New York USA; 17 Department of Neuroscience, Icahn School of Medicine at Mount Sinai, New York, New York USA; 18 Center for Genomic & Computational Biology, Duke University, Durham, North Carolina USA; 19 Division of Medical Genetics, Department of Pediatrics, Duke University, Durham, North Carolina USA; 20 Department of Psychiatry, University of Pittsburgh School of Medicine, Pittsburgh, Pennsylvania USA; 21 Department of Genetics and Genomic Sciences, Icahn School of Medicine at Mount Sinai, New York, New York USA; 22 Laboratory of Neurogenomic Biomarkers, Centre for Integrative Biology (CIBIO), University of Trento, Trento, Italy; 23 Neuropsychiatry Section, Department of Psychiatry, Perelman School of Medicine, University of Pennsylvania, Philadelphia, Pennsylvania USA; 24 Neuropsychiatric Signaling Program, Department of Psychiatry, Perelman School of Medicine, University of Pennsylvania, Philadelphia, Pennsylvania USA; 25 Department of Biomedicine, Aarhus University, Aarhus, Denmark; 26 Human Brain Collection Core, National Institutes of Health, NIMH, Bethesda, Maryland USA; 27 Department of Mathematics, University of Trento, Trento, Italy; 28 National Institute of Mental Health, Bethesda, Maryland USA; 29 Psychiatry, JJ Peters VA Medical Center, Bronx, New York, USA; 30 Department of Medicine, Psychiatry and Biomedical Informatics, Vanderbilt Genetics Institute, Vanderbilt University Medical Center, Nashville, Tennessee USA; 31 Department of Genetics, University of North Carolina at Chapel Hill, Chapel Hill, North Carolina USA; 32 Department of Biostatistics, University of Pittsburgh, Pittsburgh, Pennsylvania USA; 33 AbbVie Inc, Chicago, IL USA; 34 Helmholtz Zentrum München, Neuherberg, Germany; 35 Rush Alzheimer’s Disease Center, Rush University Medical Center, Chicago, IL USA; 36 Duke University, Durham, NC USA; 37 Nuffield Department of Medicine, Oxford University, Headington, UK; 38 Foundation for the National Institutes of Health (FNIH), Bethesda, MD USA; 39 Biogen Inc, Cambridge, MA USA; 40 Emory University, Atlanta, GA USA; 41 Department of Genetics, Harvard Medical School, Boston, MA USA; 42 Institute for Systems Biology, Seattle, WA USA; 43 Neurology, Icahn School of Medicine at Mount Sinai, New York, NY USA; 44 Picower Institute, Massachusetts Institute of Technology, Cambridge, MA USA; 45 Department of Neuroscience, University of Florida, Gainesville, FL USA; 46 GlaxoSmithKline, Brentford, UK; 47 National Center for Geriatrics and Gerontology, Nagoya, Japan; 48 Baylor College of Medicine, Houston, TX USA; 49 New York Stem Cell Foundation, New York, NY USA; 50 Brigham & Women’s Hospital, Boston, MA USA; 51 Pacific Northwest National Laboratory, Richland, WA USA; 52 Indiana University School of Medicine, Indianapolis, IN USA; 53 Broad Institute, Cambridge, MA USA

**Keywords:** Gene regulation, Quantitative trait, Gene expression

## Abstract

The availability of high-quality RNA-sequencing and genotyping data of post-mortem brain collections from consortia such as CommonMind Consortium (CMC) and the Accelerating Medicines Partnership for Alzheimer’s Disease (AMP-AD) Consortium enable the generation of a large-scale brain *cis-*eQTL meta-analysis. Here we generate cerebral cortical eQTL from 1433 samples available from four cohorts (identifying >4.1 million significant eQTL for >18,000 genes), as well as cerebellar eQTL from 261 samples (identifying 874,836 significant eQTL for >10,000 genes). We find substantially improved power in the meta-analysis over individual cohort analyses, particularly in comparison to the Genotype-Tissue Expression (GTEx) Project eQTL. Additionally, we observed differences in eQTL patterns between cerebral and cerebellar brain regions. We provide these brain eQTL as a resource for use by the research community. As a proof of principle for their utility, we apply a colocalization analysis to identify genes underlying the GWAS association peaks for schizophrenia and identify a potentially novel gene colocalization with lncRNA RP11-677M14.2 (posterior probability of colocalization 0.975).

## Introduction

Defining the landscape of genetic regulation of gene expression in a tissue-specific manner is useful for understanding both normal gene regulation and how variation in gene expression can alter disease risk. In the latter case, a variety of approaches now leverage the association between genetic variants and gene expression changes, including colocalization analysis^1–7^, transcriptome-wide association studies (TWAS)^8,9^, and gene regulatory network inference^10–16^.

There has been a relative lack of expression quantitative trait loci (eQTL) studies from the brain. Because of the more accessible nature of tissues such as blood or lymphoblastoid cell lines (LCLs), much of the large-scale identification of expression quantitative trait loci (eQTL) has occurred in these tissues^17–20^. For most other tissues, obtaining samples for RNA sequencing (RNA-seq) requires invasive biopsy, and brain tissues are typically only available in post-mortem brain samples. One effort, the Genotype-Tissue Expression (GTEx) project^21,22^, has profiled a broad range of tissues (42 distinct) for eQTL discovery, however, samples sizes in brain have been small (typically 100–150). Recently, efforts to understand gene expression changes in neuropsychiatric^23–26^ and neurodegenerative diseases^27–34^ have generated brain RNA-seq from disease and normal tissue, as well as genome-wide genotypes. These analyses have found little evidence for widespread disease-specific eQTL, as well as high cross-cohort overlap^24,35^, meaning that most eQTL detected are disease-condition independent. This implies that meta-analysis across disease-based cohorts will capture eQTL which are unconfounded by disease state despite differences in disease ascertainment of the samples, and leverages thousands of available samples to produce a well-powered brain eQTL resource for use in downstream research.

Here we generate a public eQTL resource from cerebral cortical tissue using 1433 samples from 4 cohorts from the CommonMind Consortium (CMC)^23,24,26^ and the Accelerating Medicines Partnership for Alzheimer’s Disease (AMP-AD) Consortium^30,31^, as well as from cerebellum using 261 samples from AMP-AD. We show that eQTL discovered in GTEx, which consists of control individuals (without disease) only, are replicated in this larger brain eQTL resource. We further show widespread differences in regulation between cerebral cortex and cerebellum. To demonstrate one example of the utility of these data, we apply a colocalization analysis, which seeks to identify expression traits whose eQTL association pattern appears to co-occur at the same loci as the clinical trait association, to identify putative genes underlying the GWAS association peaks for schizophrenia^36^.

## Results

We generated eQTL from the publicly available AMP-AD (ROSMAP^27,28,35,37^, Mayo RNAseq^29,38–40^) and CMC^23,24,26^ (MSSM-Penn-Pitt^24,26^, HBCC^26^) cohorts with available genotypes and RNA-seq data, using a common analysis pipeline (Supplementary Table 1) (https://www.synapse.org/#!Synapse:syn17015233). Analyses proceeded separately by cohort. Briefly, the RNA-seq data were normalized for gene length and GC content prior to adjustment for clinical confounders, processing batch information, and hidden confounders using Surrogate Variable Analysis (SVA)^41^. Genes having at least 1 count per million (CPM) in at least 50% of samples were retained for downstream analysis (Supplementary Table 2). Genotypes were imputed to the Haplotype Reference Consortium (HRC) reference panel^42^. eQTL were generated adjusting for diagnosis (AD, control, other for AMP-AD cohorts and schizophrenia, control, other for CMC cohorts) and principal components of ancestry separately for ROSMAP, Mayo temporal cortex (TCX), Mayo cerebellum (CER), MSSM-Penn-Pitt, and HBCC. For HBCC, which had a small number of samples derived from infant and adolescents, we excluded samples with age-of-death less than 18, to limit heterogeneity due to differences between the mature and developing brain.

**Fig. 1:**
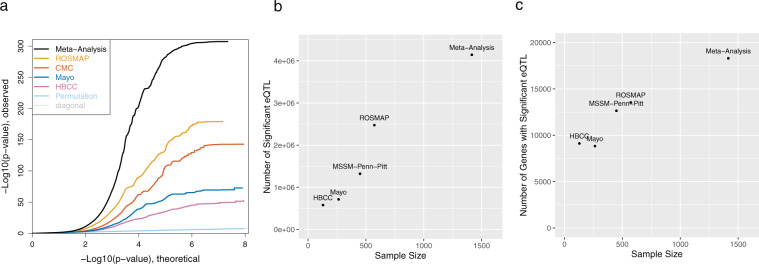
eQTL meta-analysis discovers more eQTL than individual cohorts. (**a**) Quantile-quantile plot of eQTL from individual cohorts as well as the meta-analysis of the true (black) and permuted (light blue) data. Number of significant eQTL (SNP-gene pairs) (**b**) or genes with significant eQTL (**c**) as a function of cohort size.

We then performed a meta-analysis using the eQTL from cortical brain regions from the individual cohorts (dorsolateral prefrontal cortex (DLPFC) from ROSMAP, MSSM-Penn-Pitt, and HBCC and TCX from Mayo). The meta-analysis identifies substantially more eQTL than the individual cohorts (Table 1, Fig. 1). There is a strong relationship between the sample size in the individual cohorts and meta-analysis and the number of significant eQTL and genes with eQTL (Fig. 1b,c). Notably, the meta-analysis identified significant eQTL (at FDR ≤ 0.05) in >1000 genes for which no eQTL were observed in any individual cohort. Additionally, we find significant eQTL for 18,295 (18,433 when considering markers with minor allele frequency (MAF) down to 1%) of the 19,392 genes included in the analysis.

**Table 1:** eQTL results from individual cohorts and meta-analysis.

Cohort	Cis eQTL*	Unique Genes with Signif. eQTL	eQTL (Genes) not Present in GTEx**	Meta-eQTL (Genes) not found in Cohort	Genes w/o eQTL in CMC, ROSMAP, HBCC or Mayo TCX
ROSMAP	2,472,838	13,543	2,205,025 (7,829)	2,103,711 (5,079)	
Mayo TCX	712,401	8,838	480,507 (4,439)	3,485,381 (9,591)	
CMC	1,322,680	12,641	1,062,830 (7,206)	2,948,886 (5,897)	
HBCC	577,512	9,112	379,732 (4,645)	3,614,448 (9,337)	
Meta-Analysis***	**4,142,776**	**18,295**	**3,800,208 (11,395)**		**1,042**

*SNP-gene pairs with MAF ≥ 0.02 and FDR ≤ 0.05.

**GTEx (v7) Anterior Cingulate Cortex, Cortex or Frontal Cortex.

***Additional 36,352 (138) eQTL (Genes) with 0.01 ≤ MAF < 0.02.

We then compared our cortical eQTL to those from GTEx (v7)^21^, which is the most comprehensive brain eQTL database available in terms of number of available brain tissues (Table 1, Table 2). Due to the substantially larger power in these data, we find >3.8 million eQTL not identified in GTEx cortical regions (Anterior Cingulate Cortex, Cortex or Frontal Cortex) and we find eQTL for >11,000 genes with no eQTL in these cortical regions in GTEx. While GTEx employs a stricter approach to the control of false discovery rate (FDR), we find that re-analysis of the GTEx cortical regions using an approach similar to ours (see Methods) did not account for the number of eQTL and genes with eQTL discovered in this analysis, but not in GTEx (3,619,693 and 6,866 for eQTL and genes, respectively, when using the less conservative approach). Next, we evaluated the replication within our cortical and cerebellar eQTL of the region specific eQTL identified in GTEx. The cortical eQTL generated through the current analyses strongly replicate the eQTL available through GTEx, not only for cortical regions, but for all brain regions including cervical spinal cord (Table 2). Interestingly, the replication in these cortical eQTL of eQTL derived from the two GTEx cerebellar brain regions (cerebellum and cerebellar hemisphere) is consistently lower than for other brain regions represented in GTEx. However, replication of GTEx cerebellar eQTL is high when compared to the cerebellar eQTL generated in this analysis from the Mayo Clinic CER samples. We also performed the reverse comparison, by examining the replication of our eQTL in those region-specific eQTL identified in GTEx. Unsurprisingly, the replication levels were substantially lower, due to the lower power in the GTEx analyses. Replication rates were not substantially changed by using GTEx eQTL discovered using our less conservative approach.

**Table 2:** Replication rates between GTEx and publicly available eQTL from these analyses.

GTEx Brain Region	# eQTL	Replication (π_1_) of GTEx eQTL				Replication (π_1_) of AMP-AD eQTL in GTEx			
		Meta-Analysis	ROSMAP	Mayo TCX	Mayo CER	Meta-Analysis	ROSMAP	Mayo TCX	Mayo CER
Amygdala	158,270	0.974	0.987	0.986	0.916	0.356	0.421	0.701	0.555
Anterior Cingulate Cortex (BA24)	291,898	0.976	0.971	0.962	0.903	0.445	0.516	0.772	0.592
Caudate Basal Ganglia	435,939	0.966	0.961	0.935	0.881	0.488	0.544	0.781	0.636
Cerebellar Hemisphere	575,583	0.896	0.897	0.781	0.962	0.458	0.509	0.719	0.831
Cerebellum	819,435	0.893	0.890	0.711	0.936	0.526	0.577	0.761	0.863
Cortex	478,903	0.980	0.973	0.941	0.892	0.547	0.604	0.834	0.659
Frontal Cortex (BA9)	342,988	0.974	0.967	0.963	0.897	0.489	0.547	0.792	0.632
Hippocampus	221,876	0.967	0.970	0.937	0.905	0.404	0.462	0.710	0.570
Hypothalamus	227,808	0.974	0.966	0.960	0.913	0.392	0.436	0.719	0.580
Nucleus Accumbens Basal Ganglia	376,390	0.958	0.952	0.781	0.894	0.453	0.514	0.761	0.622
Putamen Basal Ganglia	293,880	0.966	0.974	0.930	0.890	0.433	0.493	0.764	0.591
Spinal Cord Cervical (c-1)	175,702	0.940	0.952	0.918	0.862	0.330	0.394	0.640	0.514
Substantia Nigra	120,582	0.964	0.951	0.960	0.928	0.297	0.372	0.635	0.479

Additionally, we compared our eQTL to a publically available fetal brain eQTL resource^43^ and found good replication of these eQTL as well (estimated replication rate π_1_ = 0.909 for the cortical meta-analysis, and π_1_ = 0.861 for cerebellum), though somewhat lower than the replication observed in the GTEx cohorts, which are comprised of adult-derived samples.

Finally, as a proof of concept, we performed a colocalization analysis between our eQTL meta-analysis and the Psychiatric Genomics Consortium (PGC) v2 schizophrenia GWAS summary statistics^36^. Seventeen genes showed posterior probability of colocalization using coloc^7^ (PP(H_4_)) > 0.7 (Table 3), with 3 showing PP(H_4_) > 0.95 (FURIN, ZNF823, RP11-677M14.2). FURIN, having previously identified as a candidate through colocalization^24^ has recently been shown to reduce brain-derived neurotrophic factor (BDNF) maturation and secretion when inhibited by miR-338-3p^44^. ZNF823 has been identified in previous colocalization analyses^45,46^. RP11-677M14.2, a lncRNA located inside NRGN, while not previously identified through colocalization analysis, has been shown to be down-regulated in the amygdala of schizophrenia patients^47^. Noteably, NRGN does not appear to show eQTL colocalization (PP(H_4_) = 0.006), instead showing strong evidence for the eQTL and GWAS associations occurring independently (PP(H_3_) = 0.994). Two additional strong colocalizations THOC7 (PP(H_4_) = 0.943) and FAM85B (PP(H_4_) = 0.948) show other potential candidates in the region (Supplementary Table 3). At the THOC7 locus, the competing gene, C3orf49 shows slightly lower strength for colocalization (PP(H_4_) = 0.820), and the associations do not appear to be independent (R^2^ between best SNPs = 0.979). At the FAM85B locus, the competing pseudo gene FAM86B3P shows substantially lower evidence for colocalization (PP(H_4_) = 0.513) and in this case too, the associations appear to be non-independent (R^2^ = 0.902).

**Table 3:** Top colocalized genes as inferred between meta-analysis eQTL and the PGC2 schizophrenia GWAS.

Gene	eQTL Peak Location*			N_SNPs_	Min(p-value)		Best Colocalized SNP**	Posterior Probability of Colocalization	Additional Candidates at this Locus***
	Chr	Start	End		GWAS	eQTL			
RERE	1	7412645	9877280	4905	2.72E-09	2.28E-21	rs301792	0.730	
PTPRU	1	28563084	30653243	4099	1.28E-09	4.42E-10	rs1498232	0.890	
FOXN2	2	47542228	49606348	5437	1.66E-06	8.85E-45	rs79073127	0.782	
C3orf49	3	62805378	64834213	4906	2.58E-08	1.10E-13	rs832187	0.820	Yes
THOC7	3	62819766	64848612	4886	2.58E-08	2.21E-39	rs832190	0.943	Yes
TBC1D19	4	25578209	27755954	4432	7.44E-07	7.85E-07	rs6825268	0.900	
CLCN3	4	169533866	171644821	4956	1.02E-08	1.91E-09	rs10520163	0.768	
PPP1R18	6	29644275	31655438	1055	1.16E-19	5.52E-07	rs2523607	0.827	Yes
LINC00222	6	108073451	110091064	3600	3.37E-08	1.63E-07	rs9398171	0.852	
FAM85B	8	8089567	9084121	3725	2.03E-08	5.63E-31	rs2980439	0.948	Yes
ENDOG	9	130581300	132584048	3294	1.92E-06	6.90E-62	rs6478854	0.721	Yes
RP11-677M14.2	11	123614560	125616016	5253	3.68E-12	1.50E-09	rs55661361	0.975	
FURIN	15	90414642	92426654	4767	2.30E-12	1.29E-20	rs4702	0.999	
CNOT1	16	57553885	59662867	5364	1.15E-08	1.16E-06	rs12325245	0.862	
ELAC2	17	11896953	13921426	5651	2.84E-06	3.90E-106	rs1044564	0.856	
ZNF823	19	10832190	12840037	3844	1.57E-06	5.38E-10	rs3095917	0.961	
PTK6	20	61160251	62960229	4527	4.03E-08	1.09E-26	rs427230	0.897	

^*^eQTL Peak Location is ±1 Mb around the gene location.

^**^SNP showing the highest PP(H_4_) between the gene and GWAS trait.

^***^At Posterior Probability of Colocalization ≥ 0.5.

## Discussion

Using resources generated in the AMP-AD and CMC consortia, we have generated a well-powered brain eQTL resource for use by the scientific community. Unsurprisingly, we see a strong relationship between the number of significant eQTL, as well as genes with significant eQTL, and sample size using analyses from the individual cohorts as well as the meta-analysis of those cohorts. This result has previously been shown for lower sample sizes^21^. We also show higher replication of GTEx eQTL in the meta-analysis relative to the individual cohorts. These conclusions appear to be independent of methodological differences between our analysis and the one done by GTEx.

Notably, we find significant eQTL for nearly every gene in our analysis, which include all but very lowly expressed genes (less than 1 cpm in more than 50% of samples). The wide discovery of eQTL is potentially beneficial for analyses utilizing these results, such as colocalization analysis or TWAS imputation, because more genes with significant eQTL means more genes can be evaluated with these approaches. Because we have discovered eQTL for most genes, further increasing sample size will not substantially increase the number of genes with significant eQTL. However it is likely that the number of significant eQTL associations within each gene would continue to increase, which may include additional associations tagging the same regulator or independent associations tagging weaker regulators, along with the accuracy of estimated effect sizes. This will result in a more accurate landscape of regulatory association, which will improve the ability to fine-map causal regions, and colocalize eQTL signal with clinical traits of interest. Thus, it will be valuable to continue to update this meta-analysis with additional data from these consortia and other resources as they become available, and continue to improve this resource as future data permits. Future work may also focus on using well-powered analysis to study the landscape of causal variation and co-variation in gene regulation.

We found distinct eQTL patterns across cerebral cortical and cerebellar brain regions in our resource. Specifically, comparison of eQTL from our resource with those from GTEx shows high replication for the majority of brain regions. However, cerebellar regions show consistently lower replication with the cerebral cortical eQTL generated here. In contrast, the cerebellar eQTL generated from the Mayo Clinic study replicate GTEx cerebellar eQTL at a substantially higher rate, suggesting a different pattern of regulatory variation affecting expression in cerebellum versus other brain regions. Indeed, epigenomic analyses show substantial differences between cerebellar and cerebral cortical regions^48–51^, particularly in methylation patterns, which could drive different eQTL association patterns. This is further corroborated by the observation of substantial coexpression differences between cerebellar and other brain regions^52^. These effects could be due to differences in cell type composition, with cerebellar regions consisting of substantially more neurons than other brain regions^53^. This is supported by a gene enrichment analysis of genes showing different eQTL association patterns between cerebellum and cortex, which showed that many of the top gene sets were neuron or signaling related (Supplementary Table 4). One recent report suggests that there are also widespread differences in histone modifications within cell types derived from cerebellar and cortical regions^54^, though this effect had not been noted in other studies. In particular, Ma *et al*.^54^ observed that both neuronal and non-neuronal cell types show differing histone modifications across tissue of origin. Further work is necessary to confirm this finding and to develop models to deconvolve the cell-type specific regulatory effects in different brain regions^55–57^, however our analysis demonstrates that this meta-analysis is representative of eQTL across the majority of brain regions, with the exception of cerebellum. Future meta-analytic analyses may also cast a wider net in terms of brain regions included.

The replication of fetal eQTL, while significant, is somewhat lower than the replication of adult eQTL represented in GTEx. This may be due to multiple factors. The fetal eQTL analysis was generated from brain homogenate, rather than dissected brain regions, though the lower replication likely also reflects broad transcriptional differences between developing and mature brain^58^. These transcriptional differences may also explain why we find substantially more eQTL than a recently published, similarly sized eQTL analysis which uses samples from across developmental and adult timepoints^25^, and why this meta-analysis shows higher replication of GTEx eQTL.

Previous studies report a lack of widespread disease-specific eQTL observed in schizophrenia (CMC)^24^ and Alzheimer’s (ROSMAP)^35^. In accordance, we find a strong overlap among eQTL across disparate disease samples, particularly those with neuropsychiatric and neurodegenerative disorders, as well as normal individuals from these and other cohorts such as GTEx^24,35^. This suggests that disease-specific eQTL, if they exist, are likely few in number and/or small in effect size, relative to condition-independent eQTL in general. If they do exist, disease-specific eQTL discovery may be successful in more targeted analyses or with larger sample sizes or meta-analyses, but was not explored for the purpose of this general resource. Thus, the heterogeneous samples derived from different disease-based cohorts can be meta-analyzed to create a general-purpose brain eQTL resource representing adult gene regulation, despite comprising samples with different disease backgrounds, along with normal controls. Therefore, these eQTL will be useful both within and outside these specific disease contexts. For example, since these eQTL are not disease specific they may be used to understand healthy gene expression regulation in the brain, as well as to infer colocalization of eQTL signatures with disease risk for any disease whose tissue etiology is from the brain, since these signatures are reflective of normal brain regulation. It should be stated that while many eQTL are not disease specific, i.e. they are identified under various central nervous system (CNS) disease diagnoses and in control brains, they may still contribute to common CNS diseases as previously demonstrated^24,32–34,45,46^. While we have demonstrated a proof-of-concept colocalization analysis with a previously published schizophrenia GWAS, these eQTL are a broadly useful resource for studying neuropsychiatric and neurodegenerative disorders, as well as for understanding the landscape of gene regulation in brain.

## Methods

### RNA-seq Re-alignment

For the CMC studies (MSSM-Penn-Pitt, HBCC), RNA-seq reads were aligned to GRCh37 with STAR v2.4.0g1^59^ from the original FASTQ files. Uniquely mapping reads overlapping genes were counted with featureCounts v1.5.2^60^ using annotations from ENSEMBL v75.

For the AMP-AD studies (ROSMAP, Mayo RNAseq), Picard v2.2.4 (https://broadinstitute.github.io/picard/) was used to generate FASTQ files from the available BAM files, using the Picard SamToFastq function. Picard SortSam was first applied to ensure that R1 and R2 reads were correctly ordered in the intermediate SAM file before converting to FASTQ. The converted FASTQs were aligned to the GENCODE24 (GRCh38) reference genome using STAR v2.5.1b, with twopassMode set as Basic. Gene counts were computed for each sample by STAR by setting quantMode as GeneCounts.

### RNA-seq normalization

To account for differences between samples, studies, experimental batch effects and unwanted RNA-seq-specific technical variations, we performed library normalization and covariate adjustments for each study separately using fixed/mixed effects modeling. A mixed effect model was required to jointly normalize both tissues from the Mayo cohort. All other cohorts contained only one tissue, so a fixed effect model was used. The workflow consisted of the following steps:

1. ***Gene filtering:*** Out of ~56 K aligned and quantified genes, only genes showing at least modest expression were used in this analysis. Genes that were expressed more than 1 CPM (read Counts Per Million total reads) in at least 50% of samples in each tissue and diagnosis category were retained for analysis. Additionally, genes with available gene length and percentage GC content from BioMart December 2016 archive were subselected from the above list. This resulted in approximately 14 K to 16 K genes in each study.
2. ***Calculation of normalized expression values:*** Sequencing reads were then normalized in two steps. First, conditional quantile normalization (CQN)^61^ was applied to account for variations in gene length and GC content. In the second step, the confidence of sampling abundance was estimated using a weighted linear model using the voom-limma package in bioconductor^62,63^. The normalized observed read counts, along with the corresponding weights, were used in the following steps.
3. ***Outlier detection:*** Based on normalized log2(CPM) of expression values, outlier samples were detected using principal component analysis (PCA)^64,65^ and hierarchical clustering. Samples identified as outliers using both the above methods were removed from further analysis.
4. ***Covariate imputation:*** Before identifying associated covariates, important missing covariates were imputed. Principally, post-mortem interval (PMI), or the latency between death and tissue collection, which is frequently an important covariate for the analysis of gene expression from post-mortem brain tissue, was imputed for a portion of samples in Mayo RNAseq data for which true values were unavailable. Genomic predictors of PMI were estimated using ROSMAP and MSSM (an additional RNA-seq study available through AMP-AD) samples and were used to impute missing values as necessary.
5. ***Covariate identification:*** Normalized log2(CPM) counts were then explored to determine which known covariates (both biological and technical) should be adjusted. Except for the HBCC study, we used a stepwise (weighted) fixed/mixed effect regression modeling approach to select the relevant covariates having a significant association with gene expression. Here, covariates were sequentially added to the model if they were significantly associated with any of the top principal components, explaining more than 1% of variance of expression residuals. For HBCC, we used a model selection based on Bayesian information criteria (BIC) to identify the covariates that improve the model in more than 50% of genes.
6. ***SVA adjustments:*** After identifying the relevant known confounders, hidden-confounders were identified using the Surrogate Variable Analysis (SVA)^41^. We used a similar approach as previously defined^24^ to find the number of surrogate variables (SVs), which is more conservative than the default method provided by the SVA package in R^66^. The basic idea of this approach is that for an eigenvector decomposition of permuted residuals each eigenvalue should explain an equal amount of the variation. By the nature of eigenvalues, however, there will always be at least one that exceeds the expected value. Thus, from a series of 100 permutations of residuals (white noise) we identified the number of covariates as shown in Supplementary Table 1. We applied the “irw” (iterative re-weighting) version of SVA to the normalized gene expression matrix, along with the covariate model described above to obtain residual gene expression for eQTL analysis.
7. ***Covariate adjustments:*** We performed a variant of fixed/mixed effect linear regression, choosing mixed-effect models when multiple tissues or samples, were available per individual, as shown here: gene expression ~ Diagnosis + Sex + covariates + (1|Donor), where each gene was linearly regressed independently. Here Donor (individual) was modeled as a random effect when multiple tissues from the same individual were present. Observation weights (if any) were calculated using the voom-limma^62,63^ pipeline, which has a net effect of up-weighting observations with inferred higher precision in the linear model fitting process to adjust for the mean-variance relationship in RNA-seq data. The diagnosis component was then added back to the residuals to generate covariate-adjusted expression for eQTL analysis.

This workflow was applied separately for each study. For the AMP-AD studies, gene locations were lifted over to GRCh37 for comparison with the genotype imputation panel (described below). For HBCC, samples with age <18 were excluded prior to analysis. Supplementary Table 1 shows the covariates and surrogate variables identified in each study.

### AD diagnosis harmonization

Prior to RNA-seq normalization, we harmonized the LOAD definition across AMP-AD studies. AD controls were defined as patients with a low burden of plaques and tangles, as well as lack of evidence of cognitive impairment. For the ROSMAP study, we defined AD cases to be individuals with a Braak^67^ greater than or equal to 4, CERAD score^68^ less than or equal to 2, and a cognitive diagnosis of probable AD with no other causes (cogdx = 4), and controls to be individuals with Braak less than or equal to 3, CERAD score greater than or equal to 3, and cognitive diagnosis of ‘no cognitive impairment’ (cogdx = 1). For the Mayo Clinic study, we defined disease status based on neuropathology, where individuals with Braak score greater than or equal to 4 were defined to be AD cases, and individuals with Braak less than or equal to 3 were defined to be controls. Individuals not meeting “AD case” or “control” criteria were retained for analysis, and were categorized as “other” for the purposes of RNA-seq adjustment.

### Genotype QC and imputation

Genotype QC was performed using PLINK v1.9^69^. Markers with zero alternate alleles, genotyping call rate ≤ 0.98, Hardy-Weinberg *p*-value < 5e−5 were removed, as well as individuals with genotyping call rate < 0.90. Samples were then imputed to HRC (Version r1.1 2016)^42^ as follows: marker positions were lifted-over to GRCh37, if necessary. Markers were then aligned to the HRC loci using HRC-1000G-check-bim-v4.2 (http://www.well.ox.ac.uk/~wrayner/tools/), which checks the strand, alleles, position, reference/alternate allele assignments and frequencies of the markers, removing A/T & G/C single nucleotide polymorphisms (SNPs) with minor allele frequency (MAF) > 0.4, SNPs with differing alleles, SNPs with > 0.2 allele frequency difference between the genotyped samples and the HRC samples, and SNPs not in the reference panel. Imputation was performed via the Michigan Imputation Server^70^ using the Eagle v2.3^71^ phasing algorithm. Imputation was done separately by cohort and by chip within cohort, and markers with R^2^ ≥ 0.7 and minor allele frequency (MAF) ≥ 0.01 (within cohort) were retained for analysis.

### Genetic ancestry inference

GEMTOOLs^72^ was used to infer ancestry and compute ancestry components separately by cohort. The GEMTOOLs algorithm uses a significance test to estimate the number of eigenvectors (ancestry components) necessary to represent the variability in the data^73^. For each cohort, we used the top components as estimated by the GEMTOOLs algorithm, which resulted in some variation in the number of components selected. For MSSM-Penn-Pitt and HBCC, which are multi-ethnic cohorts, only Caucasian samples were retained for eQTL analysis.

### eQTL analysis

eQTL were generated separately in each cohort and tissue using MatrixEQTL^74^ adjusting for harmonized Diagnosis and inferred Ancestry components using “cis” gene-marker comparisons: Expression ~ Genotype + Diagnosis + PC_1_ + … + PC_n,_, where PC_k_ is the k^th^ ancestry component, using Expression variables which were previously covariate adjusted as described above. Here we define “cis” as ± 1 MB around the gene, and GRCh37 gene locations were used for consistency with the marker imputation panel. Meta-analysis was performed via fixed-effect model^75^ using an adaptation of the metareg function in the gap package in R. In order to assess potential inflation of Type 1 error, we performed 5 permutations of the gene expression values, relative to genotype and ancestry components, within diagnosis for each cohort, and repeated the regression analyses as described above. For each of the 5 iterations of permutation, a meta-analysis was then performed across the 4 cohorts. We found that Type 1 error was well controlled (Fig. 1a). Given that multiple tissues were present, we also evaluated a random-effect model, but found substantially deflated p-values (less significant) in the permutations, relative to the expected distribution, suggesting that this model is over-conservative.

### Comparison with GTEx and fetal eQTL

Full summary statistics for the GTEx v7^21^ eQTL for all available brain regions were obtained from the GTEx Portal (https://gtexportal.org/), and fetal eQTL were obtained from Figshare^76^. For each replication comparison (e.g. meta-analysis vs GTEx or meta-analysis vs. fetal eQTL), only markers and genes present in both the external eQTL and our analysis were retained for comparison. As this was done separately for GTEx and for the fetal eQTL resource, the list of genes and SNPs varies slightly for each comparison. The replication rate was estimated as the π_1_ statistic using the qvalue package^77^ in R as follows: we extracted the meta-analysis p-values for all SNP-gene pairs, which were significant in GTEx at FDR ≤ 0.05. We then applied the ‘qvalue’ command to the meta-analysis p-values to generate ${\widehat{\pi }}_{1}=1-{\widehat{\pi }}_{0}$, which corresponds to estimated proportion of non-null p-values^77^. The ‘smoother’ option was used to estimate ${\widehat{\pi }}_{0}$ as a function of the tuning parameter *λ* as it approaches 1. The variance around this estimate is relatively small (see Supplementary Figures 1 and 2 for example) and does not materially affect the observations in this manuscript.

Conversely, we estimated the replication rate of significant meta-analysis eQTL SNP-gene pairs in GTEx. Analogous methods were used to estimate all other replication rates. For the purposes of reporting the total number of eQTL not present in GTEx, and genes without eQTL in GTEx (Table 1), we have included genes and SNP-gene pairs not present in GTEx in the count, however this accounts for a relatively small proportion of the difference (472,995 eQTL and 1481 genes).

### GTEx eQTL generation

In order to verify that the observed power increase and replication imbalances were not due to methodological differences between this manuscript and those performed by GTEx, we obtained access to the GTEx v7 data, and generated eQTL for cortex, anterior cingulate cortex, and frontal cortex using our approach. We used gene expression and imputed genotypes as provided, as well as the provided covariates, which included 3 ancestry covariates, 14-15 surrogate variable covariates, sex and platform. We then repeated the comparisons with the meta-analysis described in the previous section, using a MAF cutoff of 0.03, which best appeared to control Type 1 error, as observed by permutation between genotype and gene expression, while maximizing the number of significant eQTL in the true data. Results did not change materially.

### Pathway analysis of cerebellar eQTL genes

In order to identify whether genes showing cerebellar-specific eQTL patterns showed any biological coherence, we performed a pathway analysis as follows. For genes with at least 5 significant cerebellar eQTL, we computed the Spearman correlation of effect-size between cerebellum eQTL and cortical eQTL for the loci that were significant in cerebellum. We then selected genes for which the effect-sizes did not show positive correlation (Spearman’s ρ < 0.1) between the two tissues as showing different eQTL association patterns across the gene and performed a pathway analysis with GO biological processes Fisher’s exact test. Note that due to the (power-mediated) greater detection of eQTL in cortex, we did not perform the reverse comparison. The results were relatively robust to the choice of minimum number of significant eQTL, correlation cutoff, and choice of correlation statistic (Spearman vs Pearson).

### Coloc analysis

We applied Approximate Bayes Factor colocalization (coloc.abf)^7^ from the coloc R package to the summary statistics from the PGC2 Schizophrenia GWAS^36^ downloaded from the PGC website (http://pgc.unc.edu), and the summary statistics from the eQTL meta-analysis. Each gene present in the meta-analysis was compared to the GWAS in turn, and suggestive and significant GWAS peaks with *p*-value < 5e-6 were considered for analysis.

## Supplementary information

Supplementary Figures

Supplementary Table 1

Supplementary Table 2

Supplementary Table 3

Supplementary Table 4

## Data Availability

Data for the ROSMAP^37^ and Mayo cohorts^40^ are available through the AMP-AD Knowledge Portal^31^. Data for the MSSM-Penn-Pitt and HBCC cohorts are available through the CommonMind Knowledge Portal^23^. eQTL results for the ROSMAP^78^, Mayo TCX^79^, Mayo CER^80^ and cortical meta-analysis^81^ are available through the AMP-AD Knowledge Portal. These results include SNP (location, rsid, alleles, and allele frequency) and gene (location, gene symbol, strand and biotype) information, as well as estimated effect size (beta), statistic (z), p-value, FDR, and expression-increasing allele.

## Code availability

An R package with all code for the gene expression normalization is available at https://github.com/Sage-Bionetworks/ampad-diffexp. All other analyses were generated using packages publicly available from their respective authors.
