# Supplementary material for: Large eQTL meta-analysis reveals differing patterns between cerebral cortical and cerebellar brain regions
Source: Sci Data. 2020 Oct 12;7:340. doi: 10.1038/s41597-020-00642-8 (PMC7550587; doi:10.1038/s41597-020-00642-8)
Supplement: Supplementary file 1 — Supplementary Figures [file 41597_2020_642_MOESM1_ESM.pdf]

Supplementary Figures for Sieberts et al.

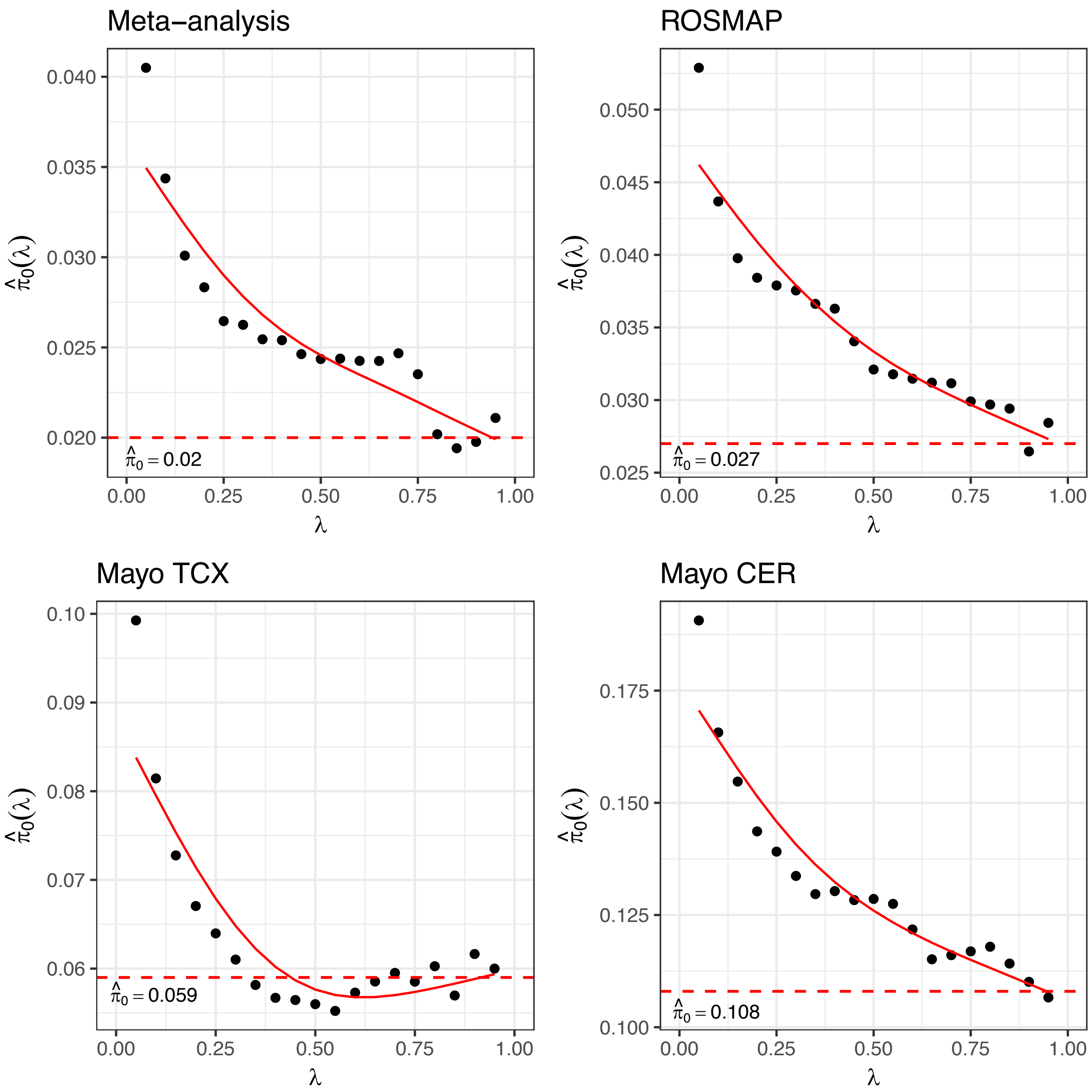

Supplementary Figure 1: $\hat{\pi}_0(\lambda)$ versus λ for the estimated replication of GTEx cortex eQTL in the (a) meta-analysis, (b) ROSMAP, (c) Mayo temporal cortex, and (d) Mayo cerebellum eQTL. Here, $\hat{\pi}_0(\lambda) = 1 - \hat{\pi}_1(\lambda)$ is a function of the tuning parameter λ. These plots demonstrate the potential range of the estimated replication rates, π_1, across choice of λ, as well as the potential error in the estimate of $\hat{\pi}_0(\lambda \to 1)$ which was reported in the manuscript.

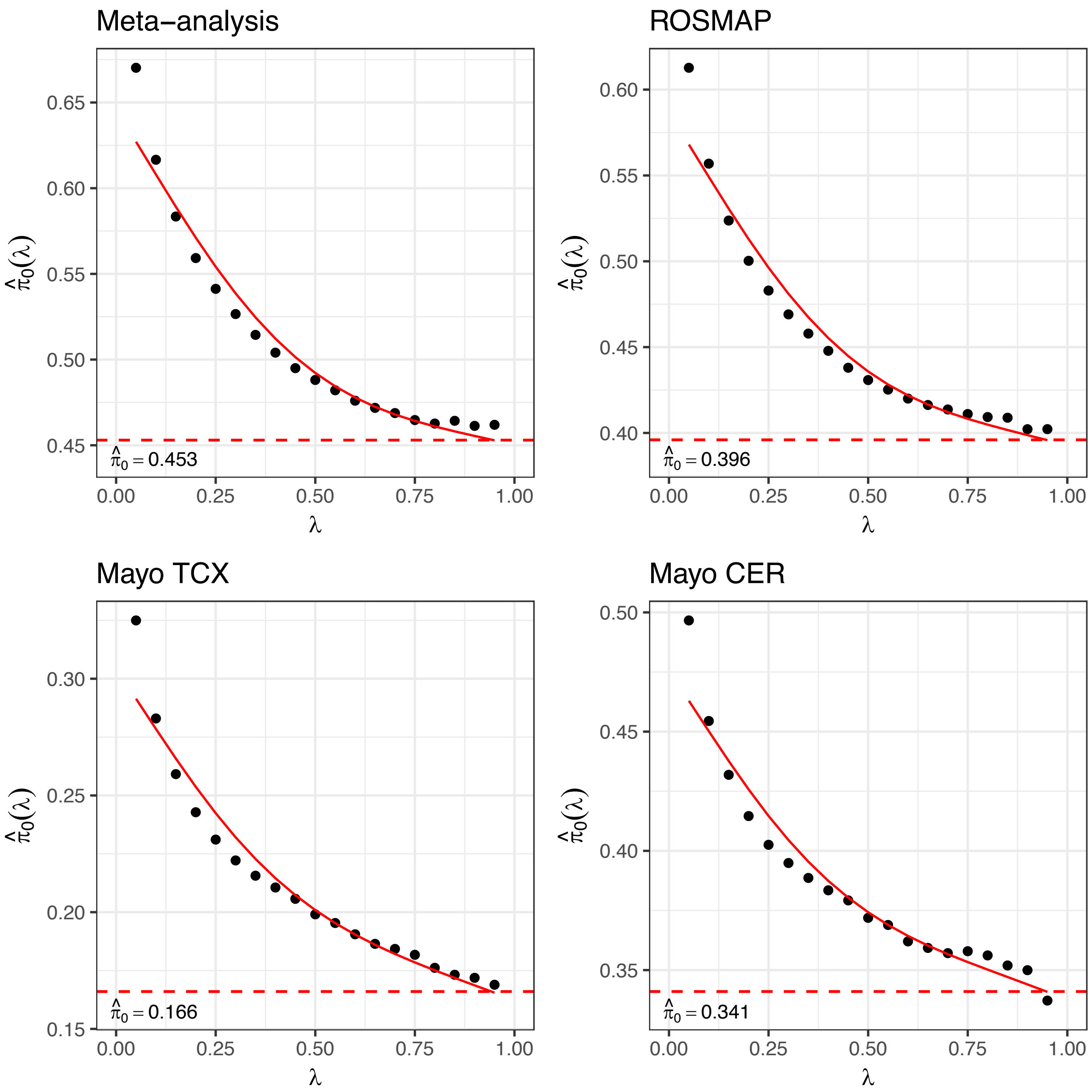

Supplementary Figure 2: $\hat{\pi}_0(\lambda)$ versus λ for the estimated replication of the (a) meta-analysis, (b) ROSMAP, (c) Mayo temporal cortex, and (d) Mayo cerebellum eQTL in GTEx cortex. Here, $\hat{\pi}_0(\lambda) = 1 - \hat{\pi}_1(\lambda)$ is a function of the tuning parameter λ. These plots demonstrate the potential range of the estimated replication rates, π_1, across choice of λ, as well as the potential error in the estimate of $\hat{\pi}_0(\lambda \rightarrow 1)$ which was reported in the manuscript.
