# Supplementary material for: Large eQTL meta-analysis reveals differing patterns between cerebral cortical and cerebellar brain regions
Source: Sci Data. 2020 Oct 12;7:340. doi: 10.1038/s41597-020-00642-8 (PMC7550587; doi:10.1038/s41597-020-00642-8)
Supplement: Supplementary file 2 — Supplementary Table 1 [file 41597_2020_642_MOESM2_ESM.docx]

Supplementary Table 1: Demographic and diagnosis data for samples used in the eQTL meta-analysis.

| **Resource** | **Cohort** | | **Diagnosis** | **N** | **Age (years)** | **% Male** |
| --- | --- | --- | --- | --- | --- | --- |
| AMP-AD Consortium | ROSMAP | | AD | 138 | 88.4±2.9 | 28.3 |
|  |  |  | Control | 82 | 83.4±5.8 | 45.1 |
|  |  |  | Other | 353 | 86.8±4.2 | 38.0 |
|  | Mayo | | AD | 80 | 82.6±7.7 | 38.8 |
|  |  |  | Control | 71 | 82.7±8.5 | 50.7 |
|  |  |  | Other | 111 | 77.1±7.7 | 55.0 |
| CommonMind Consortium | MSSM-Penn-Pitt | Mount Sinai (MSSM) | Schizophrenia | 110 | 74.1±12.0 | 68.2 |
|  |  |  | Bipolar/Other | 18 | 60.9±16.1 | 38.9 |
|  |  |  | Control | 105 | 76.5±17.8 | 49.5 |
|  |  | University of Pennsylvania | Schizophrenia | 47 | 81.0±6.9 | 40.4 |
|  |  |  | Control | 25 | 67.7±17.1 | 52.0 |
|  |  | University of Pittsburgh | Schizophrenia | 39 | 50.0±12.4 | 82.1 |
|  |  |  | Bipolar | 34 | 46.0±12.1 | 58.8 |
|  |  |  | Control | 71 | 49.2±13.8 | 70.4 |
|  | HBCC | | Schizophrenia | 35 | 48.6±15.1 | 71.4 |
|  |  |  | Bipolar/Other | 47 | 43.7±15.2 | 70.2 |
|  |  |  | Control | 67 | 43.8±16.6 | 85.1 |
