# Supplementary material for: Large eQTL meta-analysis reveals differing patterns between cerebral cortical and cerebellar brain regions
Source: Sci Data. 2020 Oct 12;7:340. doi: 10.1038/s41597-020-00642-8 (PMC7550587; doi:10.1038/s41597-020-00642-8)
Supplement: Supplementary file 3 — Supplementary Table 2 [file 41597_2020_642_MOESM3_ESM.docx]

Supplementary Table 2: RNA normalization and modeling used to adjust RNA-seq data prior to eQTL analysis, as well as covariates used in the eQTL model.

| Source/Cohort | | Brain Region (Samples) | RNA Normalization | | | | | eQTL Model | |
| --- | --- | --- | --- | --- | --- | --- | --- | --- | --- |
|  |  |  | Fixed effects | | | Random effect | Genes after Filtering | Number of Ancestry PCs | Dx |
|  |  |  | Clinical covariates | Technical covariates | Surrogate Variables |  |  |  |  |
| AMP-AD | ROSMAP | DLPFC  (573) | Sex, PMI*, Age of Death | Batch, RIN**, PCT_CODING_BASES, PCT_INTERGENIC_BASES | 21 | - | 15,582 | 3 | AD, Control, Other |
|  | MAYO | CER (261) | Sex, PMI, Age of Death | Source, FLOWCELL, PCT_INTRONIC_BASES, RIN, PCT_INTERGENIC_BASES, PCT_CODING_BASES, PCT_RIBOSOMAL_BASES | 19 | Donor | 17,003 | 2 | AD, Control, Other |
|  |  | TCX (262) |  |  |  |  |  |  |  |
| CommonMind | MSSM-Penn-Pitt | DLPFC (449) | Institution, Sex | RIN, IntronicRate | 32 | Donor | 18,841 | 5 | Schizphrenia, Control, Other |
|  | HBCC | DLPFC (149) | Sex | RIN, IntronicRate | 13 | - | 17,250 | 5 | SCZ, Control, Other |

* Post-mortem interval (PMI)

** RNA integrity number (RIN)
